# Plasmid-Mediated Quinolone Resistance Genes and Antibiotic Residues in Wastewater and Soil Adjacent to Swine Feedlots: Potential Transfer to Agricultural Lands

**DOI:** 10.1289/ehp.1104776

**Published:** 2012-05-08

**Authors:** Juan Li, Thanh Wang, Bing Shao, Jianzhong Shen, Shaochen Wang, Yongning Wu

**Affiliations:** 1 Key Laboratory of Development and Evaluation of Chemical and Herbal Drugs for Animal Use, College of Veterinary Medicine, China Agricultural University, Beijing, China; 2 Ministry of Health Key Laboratory, China National Center for Food Safety Risk Assessment, Beijing, China; 3 Key Laboratory of Chemical Safety and Health, Chinese Center for Disease Control and Prevention, Beijing, China; 4 Research Center for Eco-Environment Science, Chinese Academy of Science, Beijing, China; 5 Beijing Key Laboratory of Food Poison Diagnostic and Traceability, Beijing Center for Disease Control and Prevention, Beijing, China; 6 College of Public Health and Family Medicine, Capital Medical University, Beijing, China

**Keywords:** agricultural soil, culture-independent method, environmental health, (fluoro)quinolones, PMQR genes, swine feedlot, wastewater

## Abstract

Background: Inappropriate use of antibiotics in swine feed could cause accelerated emergence of antibiotic resistance genes, and agricultural application of swine waste could spread antibiotic resistance genes to the surrounding environment.

Objectives: We investigated the distribution of plasmid-mediated quinolone resistance (PMQR) genes from swine feedlots and their surrounding environment.

Methods: We used a culture-independent method to identify PMQR genes and estimate their levels in wastewater from seven swine feedlot operations and corresponding wastewater-irrigated farm fields. Concentrations of (fluoro)quinolones in wastewater and soil samples were determined by ultra-performance liquid chromatography–electrospray tandem mass spectrometry.

Results: The predominant PMQR genes in both the wastewater and soil samples were *qnr*D, *qep*A, and *oqx*B, whereas *qnr*S and *oqx*A were present only in wastewater samples. Absolute concentrations of all PMQR genes combined ranged from 1.66 × 10^7^ to 4.06 × 10^8^ copies/mL in wastewater and 4.06 × 10^6^ to 9.52 × 10^7^ copies/g in soil. Concentrations of (fluoro)quinolones ranged from 4.57 to 321 ng/mL in wastewater and below detection limit to 23.4 ng/g in soil. Significant correlations were found between the relative abundance of PMQR genes and (fluoro)quinolone concentrations (*r* = 0.71, *p* = 0.005) and the relative abundance of PMQR genes in paired wastewater and agricultural soil samples (*r* = 0.91, *p* = 0.005).

Conclusions: Swine feedlot wastewater may be a source of PMQR genes that could facilitate the spread of antibiotic resistance. To our knowledge, this is the first study to examine the occurrence of PMQR genes in animal husbandry environments using a culture-independent method.

The growth of the swine-breeding industry has led to the increased use of antibiotics for therapeutic purposes and to promote growth and improve feed efficiency, including some antibiotics that are important in human clinical medicine. The use of subtherapeutic concentrations of antibiotics for nontherapeutic purposes could drive the selection of bacterial resistance in the gastrointestinal tracts of swine (Chee-Sanford et al. 2001; Mackie et al. 2006). Under chronic antimicrobial pressure, resistance may increase because of the rapid reproduction and spread of resistant strains (Silbergeld et al. 2008). Jensen et al. (2006) and Bager et al. (1997) found that the agricultural use of antibiotics had a significant effect on the prevalence of antibiotic-resistant bacteria in swine waste. A large proportion of swine waste is typically stored in open-air lagoons and subsequently applied to surrounding agricultural fields through irrigation or fertilization (Sapkota et al. 2007) although some may be discharged into surrounding rivers via drainage ditches. These activities might pose a risk to public health if they result in the spread of genetic elements encoding antibiotic resistance and the spread of unabsorbed antibiotics into the environment (Pruden et al. 2006).

Antibiotic resistance genes (ARGs) released from dead microorganisms can persist in the environment for an extended period of time (Pote et al. 2003) and spread among bacteria through vertical transfer (generation) or horizontal transfer (conjugation, transduction, transformation, and transposition). The ARGs could therefore be considered to be emerging environmental “contaminants” as defined by Pruden et al. (2006), and they have the potential to be further distributed to various environmental compartments (Agerso and Sandvang 2005; Rodriguez et al. 2006). There have been various strategies for investigating environmental ARGs. One such strategy is by a culture-independent method that analyzes DNA extracted from all the microorganisms present in environmental samples (Riesenfeld et al. 2004). This avoids bias that results from the non-culturability of a large proportion of microorganisms in standard culture conditions (Rappe and Giovannoni 2003) and from variation in the effects of environmental media on the success of culture-based techniques (Allen et al. 2010). Seyfried et al. (2010) identified tetracycline resistance (*tet*^R^) genes associated with oxytetracycline use in aquaculture facilities. They also demonstrated that Class 1 integron gene and *tet*^R^ genes (*tet*A and *tet*C) were disseminated in different aquatic environments in Jiangsu Province in China (Zhang et al. 2009). The various *tet*^R^ genes and erythromycin resistance genes found in different environmental compartments appeared to be influenced by surrounding swine feedlots (Chen et al. 2010; Wu et al. 2010). Moreover, some studies have reported that the absolute concentrations of *tet*^R^ genes were significantly correlated with the concentrations of corresponding antibiotic residues in the environment (Peak et al. 2007; Wu et al. 2010).

(Fluoro)quinolones are broad-spectrum antimicrobial agents that predominantly have been used to treat various infections in humans and animals. Their current use has been extended to employment as a growth enhancer in pigs (Danish Integrated Antimicrobial Resistance Monitoring and Research Programme 1999). The expanded usage of (fluoro)quinolones has also lead to serious cases of widespread resistance to these agents (Strahilevitz et al. 2009). Before the emergence of bacterial plasmid-mediated quinolone resistance (PMQR) genes, research on the resistance mechanisms of (fluoro)quinolones were confined to mutations of chromosomal genes coding DNA gyrase or topoisomerase IV in the quinolone resistance determining region (QRDR) (Hopkins et al. 2005). Currently, three types of PMQR genes and their variations have been more frequently reported in various bacterial pathogens around the world. These are the quinolone resistance determinant (*qnr*) genes (*qnr*A, *qnr*B, *qnr*C, *qnr*D, and *qnr*S), variant aminoglycoside acetyltransferase gene [*aac(6´)-Ib-cr*], and efflux pumps-encoding genes (*qep*A and *oqx*AB) (Cattoir et al. 2008; Hernandez et al. 2011; Strahilevitz et al. 2009). The gene *qnr*A was one of the first identified PMQR genes (Martinez-Martinez et al. 1998), and research on the PMQR genes has since expanded in environmental and health science (Robicsek et al. 2006; Strahilevitz et al. 2009). The presence of *qnr* genes may increase the selection of mutations with high-level (fluoro)quinolone resistance (Rodriguez-Martinez et al. 2007). Furthermore, PMQR genes usually combine with other resistance genes in the same plasmid, so the presence of any other antibiotics for which the plasmid confers resistance will select for quinolone resistance as well (Hernandez et al. 2011). The PMQR genes may also be horizontally transmitted among bacterial isolates of different origins (Martinez-Martinez et al. 1998; Zhao et al. 2010).

To our knowledge, only two previous publications have reported the study of the environmental occurrence of PMQR genes (Cummings et al. 2011; Kristiansson et al. 2011). In contrast to those studies, we used a culture-independent genomics study method to investigate the occurrence of PMQR genes in wastewater samples collected from swine feedlots and corresponding soil samples from nearby agricultural fields where the wastewaters were used for irrigation.

## Materials and Methods

*Sampling procedure.* Wastewater samples (about 2.5 L for each site) were collected from the effluent of seven conventional swine feedlots located in three districts of Beijing: Fangshan District (defined as F_1_-w, F_2_-w, and F_3_-w), Daxing District (D_1_-w, D_2_-w, and D_3_-w), and Shunyi District (S-w), during August 2010. These feedlot effluents are periodically used for irrigation in surrounding agricultural fields and are occasionally discharged into surrounding rivers. Before collecting the wastewater samples, samplers and sample bottles were rinsed three times with ethanol, once with sterile deionized water, and three times with the wastewater. At each site, three swine feedlot wastewater samples were collected at 1–2 hr intervals and then combined to form one composite sample for each site. The wastewater samples were stored in presterilized 500-mL amber polypropylene high-density bottles (Embalator AB, Ulricehamn, Sweden) equipped with Teflon-lined polypropylene caps.

Concurrent with the collection of wastewater samples, soil samples (about 500 g for each site) were collected from agricultural fields adjacent to the seven swine feedlots (defined as F_1_-s, F_2_-s, F_3_-s, D_1_-s, D_2_-s, D_3_-s, and S-s) using a shovel and sterilized amber plastic bags. For each site, the top 15 cm of the surface soil from three different locations were pooled to form one composite sample. For example, F_1_-w and F_1_-s are paired wastewater and soil samples from the same site; this also applies for samples from the other sample sites.

Additionally, surface river water and corresponding farm soil samples collected at sites upstream from the swine feedlots were used as control samples (and defined as being uncontaminated by wastewater from swine feedlot operations).

Sampling was kept as sterile as possible, and all samples were immediately stored in a cooler box until returned to the laboratory for immediate processing (< 12 hr).

*Sample processing and DNA extraction.* Each composite water or soil sample was divided into two aliquots under aseptic conditions. One aliquot was used for quantification of (fluoro)quinolone residues after storage at 4°C for ≤ 1 week. The other was used for quantification of PMQR genes. Each aliquot was further divided into three subsamples. Sample processing for molecular analyses was always carried out first.

Power Water DNA Kits (MO BIO Laboratories Inc., Carlsbad, CA, USA) were used to extract total DNA from each wastewater subsample after pretreatment to remove particulates via layered filtration with Whatman grade 4 qualitative filter paper (20–25 μm), Whatman grade 3 qualitative filter paper (6 μm) and glass-fiber Whatman GF/B (1 μm). Approximately 200 mL of each prefiltered wastewater subsample was immediately concentrated in duplicate using the sterile filter (0.2 μm) from the Power Water DNA Kit (MO BIO). We followed the manufacturer’s protocol for the subsequent extraction steps.

Soil subsamples were aseptically equilibrated and homogenized at room temperature. All thawed soil subsamples were passed through a 2.0-mm sieve, and about 1 g of homogenized soil was extracted in duplicate using a commercial Power Soil DNA Kit (MO BIO) in accordance with the manufacturer’s instructions.

We performed DNA extractions for each subsample in duplicate, and the duplicate extracts were then pooled to form a single composite sample for that site that was stored at –80°C until subsequent molecular analyses.

*Polymerase chain reaction (PCR) assays for PMQR genes.* Qualitative PCR assays were used to assess the presence of nine PMQR genes [*qnr*A, *qnr*B, *qnr*C, *qnr*D, *qnr*S, *qep*A, *oqx*A, *oqx*B, *aac(6*´*)-Ib-cr*] in all environmental and control subsamples. All primers were previously validated [for primer sequences, amplicon sizes, annealing temperatures, references for each sequence, and additional details regarding PCR conditions, see Supplemental Material, Table S1 (http://dx.doi.org/10.1289/ehp.1104776)]. To ensure reproducibility, two replicates for each sample were performed in parallel with a control sample in each run. To prevent false-negative results due to PCR-inhibiting substances such as humic acids, 2 ng of DNA extract from each sample that did not show amplification of each target gene was spiked with positive-control template at 10^2^ copies/μL. There was no evidence of PCR inhibition in any extracts (data not shown). An Agilent 2100 bioanalyzer (Agilent Technologies, Santa Clara, CA, USA) was used to analyze DNA fragments (Panaro et al. 2000). Chips in the DNA 7500 LabChip kit (Agilent Technologies) were loaded with PCR amplification products according to the manufacturer’s instructions, with minor modifications. Briefly, microchannels on the chips were filled by pipetting 9 μL of gel-dye mixture into the appropriate well and then forcing the mixture into the microchannels by applying pressure to the well via a 1-mL syringe. The ladder well was subsequently loaded with 5 μL of marker mixture plus 1 μL of molecular size ladder, while sample wells were loaded with 5 μL of marker mixture plus 1 μL of PCR amplification products. The marker mixture for the Agilent DNA 7500 Lab Chip contains lower and upper molecular size markers of 50 bp and 10,380 bp, respectively. After vortexing for 1 min, the chip (with 12 PCR amplification products) could be read within 30 min by the Agilent 2100 bioanalyzer.

Amplification products from each positive sample were purified with PCR quick spin™ PCR Product Purification Kit (Tiangen Biotech Co. Ltd., Beijing, China) and ligated into pGEM-T Easy Vector (Promega, Madison, WI, USA) before being cloned into *Escherichia coli* DH5α using the pEASY-T1 Simple Cloning Kit (TransGen Biotech Co. Ltd., Beijing, China). Clones containing target gene inserts were selected and confirmed by PCR. Plasmids carrying target genes were extracted and purified with the MiniBEST Plasmid Purification Kit (TaKaRa, Dalian, China) and sequenced by Invitrogen Ltd./Applied Biosystems Ltd. (Beijing, China), and the resulting sequences were compared with GenBank (http://www.ncbi.nlm.nih.gov/nuccore/) sequences for the target genes using the BLAST alignment tool (http://www.ncbi.nlm.nih.gov/blast/). These plasmids were used to generate standard curves for subsequent quantification of each gene in the subsamples as described below.

*Quantitation of PMQR genes.* The quantitative PCR (qPCR) reactions were conducted using SYBR Green I chemistry and the Bio-Rad Chromo4 real-time PCR instrument (both from Bio-Rad, Hercules, CA, USA) with the Analysis software version 3.0 (BioRad) to quantify levels of the PMQR genes and 16S rRNA in all subsamples. Sample-derived standards were diluted serially in molecular biology-grade water. The qPCR reactions were conducted in 96-well plates. Optimal qPCR conditions were determined empirically [for details, see Supplemental Material, pp. 2–3 (http://dx.doi.org/10.1289/ehp.1104776)]. DNA extracts were amplified against the 10-fold serially diluted calibration curve over seven orders of magnitude and DNA-free negative control on the same real-time PCR plate in triplicate. Standard error values of the measurements were determined from these parallel data. 16S rRNA was also quantified (Graham et al. 2011; Peak et al. 2007; Smith et al. 2004) on the same plate, using the SybrGreen (BioRad) approach. Following qPCR, melting curves for the amplicons were measured by slowly raising the temperature while monitoring fluorescence to verify that nonspecific amplification did not occur (data not shown). Matrix effects associated with extraction of DNA from environmental samples were corrected as described by Pei et al. (2006). The presence of inhibitory substances in the sample matrix was assessed by spiking the samples with defined amounts of DNA template and comparing concentration thresholds between the matrix and controls, whose difference was always < 1. The qPCR efficiencies (90–102% in this study) were examined by comparing plasmid controls and serial dilutions of selected samples, using a 16S rRNA assay as described in the Supplemental Material (pp. 2–3). There was very low inhibition in these samples (data not shown). Results from the assays were analyzed based on the slope for the qPCR calibration curve. R^2^ values were greater than 0.992 for all calibration curves.

Copy numbers of target PMQR genes were normalized to the 16S rRNA copy number (defined as relative abundances) and to 1 g for soil samples or 1 mL for wastewater samples (defined as absolute concentrations: copies per gram or copies per milliliter) to take into account any temporal variations among sites, overall extraction efficiencies, total bacterial community, and potential sample degradation (Graham et al. 2011). We used the term “levels” to describe findings that relate to both relative abundances and absolute concentrations.

*Quantitation of (fluoro)quinolones.* In this study, extraction and quantitative analysis of (fluoro)quinolone residues in wastewater and soil samples was performed according to Shao et al. (2009). The method is based on solid-phase extraction (SPE) and analysis by ultra performance liquid chromatography–tandem mass spectrometry (UPLC-MS/MS; Acquity UPLC; Waters Corporation, Milford, MA, USA). Orbifloxacin, danofloxacin, pipemidic acid, marbofloxacin, lomefloxacin, pefloxacin, enrofloxacin, norfloxacin, ciprofloxacin, and ofloxacin were purchased from Sigma (St. Louis, MO, USA) with purities > 97%. The recoveries for (fluoro)quinolones based on matrix-matched calibration ranged between 100% and 117% in aqueous solution and between 77% and 114% in soil samples, and the quantification limits were in the range of 0.2–10 pg/mL for water and 0.38–2.00 ng/g for soil samples. The analytical method is described in detail in Supplemental Material, p. 2 (http://dx.doi.org/10.1289/ehp.1104776).

*Statistical analysis.* Data analysis was conducted using SPSS Statistics version 16.0 (IBM, Armonk, NY, USA). A one-way analysis of variance test and independent-sample *t*-tests were used to compare samples with controls (the level for statistical significance was set at *p* < 0.05). Data were log-transformed when necessary to obtain a normal distribution before statistical analysis. A two-tailed Pearson’s bivariate correlation analysis was used to compare levels of total PMQR genes in soil and wastewater samples and to compare levels of total PMQR genes and (fluoro)quinolone concentrations.

## Results

*Occurrence and levels of PMQR genes.* Among the nine PMQR genes investigated, *qnr*D, *oqx*B, and *qep*A were found in all environmental samples from the target sites; *qnr*S and *oqx*A were only detected in wastewater samples; *qnr*B was found in only three wastewater samples (F_3_-w, D_2_-w, and D_3_-w); and *qnr*A, *qnr*C, and *aac(6´)-Ib-cr* were not detected at all [see Supplemental Material, Figure S1 and Table S2 (http://dx.doi.org/10.1289/ehp.1104776)]. In addition, no PMQR genes were detected in control samples. The positively identified PMQR genes were identical to the corresponding sequences deposited in the GenBank database (data not shown).

**Figure 1:**
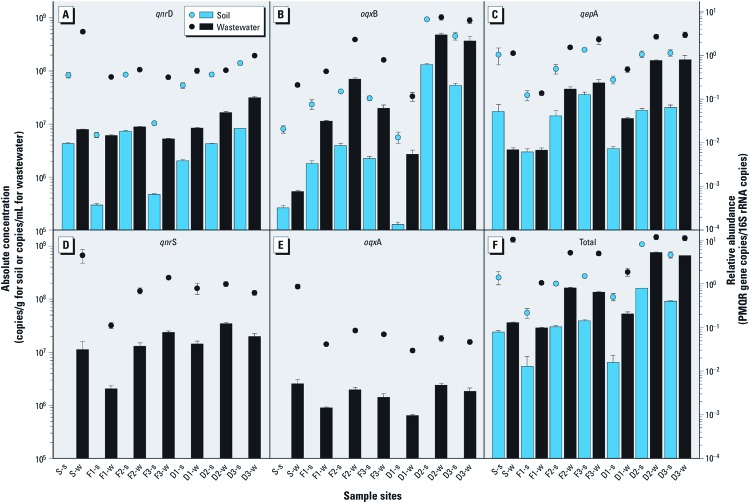
Levels of five PMQR genes among the soil and wastewater samples. (*A*) *qnr*D. (*B*) *oqx*B. (*C*) *qep*A. (*D*) *qnr*S. (*E*) *oqx*A. (*F*) Total of the five PMQR genes. Bars represent absolute concentrations and circles represent relative abundances. Values shown are mean ± SE of three analytical replicates.

Absolute concentrations and relative abundances of the five major PMQR genes (*qnr*D, *oqx*B, *qep*A, *qnr*S, and *oqx*A) are shown in Figure 1 and reported in Supplemental Material Tables S3 and S4, respectively (http://dx.doi.org/10.1289/ehp.1104776).

**Figure 2:**
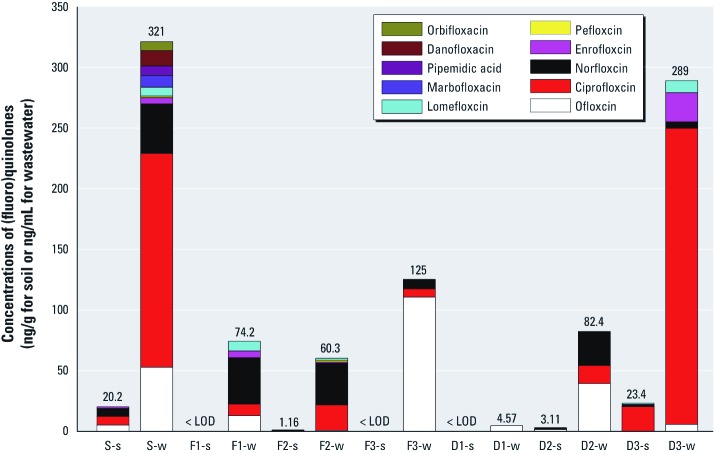
Concentrations of 10 (fluoro)quinolones (ng/mL or ng/g) in study samples. Bars represent mean concentrations of the 10 (fluoro)quinolones. Numbers above bars show the total concentration of (fluoro)quinolones in each composite sample. The concentrations of the 10 (fluoro)quinolones were < LOD in all control samples.

***Concentrations of (fluoro)quinolones.*** Ten (fluoro)quinolones were detected in the samples (orbifloxacin, danofloxacin, pipemidic acid, marbofloxacin, lomefloxacin, pefloxacin, enrofloxacin, norfloxacin, ciprofloxacin, and ofloxacin) with concentrations ranging from below the limit of detection (LOD) to 244 ng/mL in wastewater samples and 20.4 ng/g in soil samples [see Supplemental Material, Table S5 (http://dx.doi.org/10.1289/ehp.1104776)]. All (fluoro)quinolones were < LODs in all control samples. Norfloxacin was detected in all target samples with the exception of three soil samples (F_1_-s, F_3_-s, and D_1_-s) in which none of the (fluoro)quinolones was detected (Figure 2). The frequencies of detection were thereafter followed by ofloxacin, ciprofloxacin, enrofloxacin, and lomefloxacin. Other (fluoro)quinolones (marbofloxacin, pipemidic acid, danofloxacin, and orbifloxacin) were detected only in the S-w wastewater sample, whereas pefloxacin was found in S-w and F_2_-w. The average concentration of norfloxacin was 1.16 ng/g in soil and 40.6 ng/mL in wastewater samples (see Supplemental Material, Table S5). Ciprofloxacin was identified in the wastewater and soil samples from the D_3_ site at the highest concentration of any of the (fluoro)quinolones measured in wastewater (244 ± 2.04 ng/mL in D_3_-w) and soil (and 20.4 ng/g in D_3_-s) samples, respectively (see Supplemental Material, Table S5). Total (fluoro)quinolone concentrations were highest in S-w (321 ng/mL) among the wastewater samples and at the D_3_-s site (23.4 ng/g) among soils (see Supplemental Material, Table S5).

*Correlation analysis.* Significant positive correlations between paired wastewater and soil samples were observed for the relative abundances of *qep*A (*r* = 0.94, *p* = 0.001), *oqx*B (*r* = 0.96, *p* = 0.001), and total PMQR genes (sum of five PMQR genes: *qnr*D, *qep*A, *oqx*B, *qnr*S, and *oqx*A; *r* = 0.91, *p* = 0.005) but not *qnr*D (*r* = 0.56, *p* = 0.19). Absolute concentrations were significantly correlated between paired soil and water samples for *oqx*B (*r* = 0.95, *p* = 0.001) and total PMQR genes (*r* = 0.91, *p* = 0.005), but not *qep*A (*r* = 0.63, *p* = 0.13) or *qnr*D (*r* = 0.72, *p* = 0.07).

**Figure 3:**
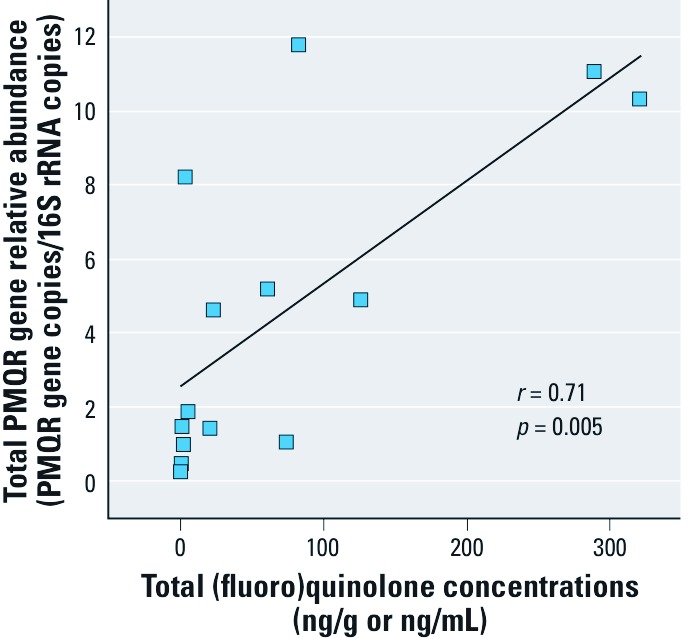
Correlation between the relative abundance of total combined PMQR genes (*qnr*D, *oqx*B, *qep*A, *qnr*S, and *oqx*A) and the total combined (fluoro)quinolone concentration (orbifloxacin, danofloxacin, pipemidic acid, marbofloxacin, lomefloxacin, pefloxacin, enrofloxacin, norfloxacin, ciprofloxacin, and ofloxacin).

The relative abundance of total PMQR genes (sum of the five genes: *qnr*D, *oqx*B, *qep*A, *qnr*S, and *oqx*A) and measured concentrations of total (fluoro)quinolones were significantly correlated (*r* = 0.71, *p* = 0.005) (Figure 3). Significant correlations also were observed for some but not all concentrations of individual (fluoro)quinolones and the relative abundance of individual PMQR genes and for total (fluoro)quinolones and four of five individual PMQR genes [see Supplemental Material, Table S6 (http://dx.doi.org/10.1289/ehp.1104776)].

## Discussion

We investigated wastewater from seven different swine feedlots and corresponding agricultural soil samples for the presence of PMQR genes. The findings indicate that *qnr*D, *oqx*B, *qep*A, *qnr*S, and *oqx*A genes were widespread in almost all of the wastewater samples. In another study, some of these PMQR genes were also found in environmental *E. coli* strains from swine (Liu et al. 2008), and *oqx*A and *oqx*B have been found in a conjugative plasmid that conferred resistance to the antibiotic olaquindox, which has been used as a swine growth enhancer (Hansen et al. 2007). Additionally, *qnr*S has been found in *E. coli* strains from swine in China (Xia et al. 2010). In a survey by Cummings et al. (2011), *qep*A and *qnr*S were commonly observed PMQR genes in microbial DNA extracted from surface sediments of the Tijuana River Estuary in San Diego County, California, USA. Less information is available on the environmental occurrence of the PMQR gene, *qnr*D, which previously has been identified in samples from humans and companion animals only (Zhu et al. 2010) but was observed in all samples in the present study. Interestingly, the *aac(6´)-Ib-cr* gene, which has been reported as the most common PMQR gene among clinical *Enterobacteriaceae* isolates (Strahilevitz et al. 2009), was absent in all samples in the present study.

Although levels of the three PMQR genes (*qnr*D, *oqx*B, and *qep*A) varied among the environmental samples, they were identified in all wastewater samples and corresponding farm soil samples. In contrast, no PMQR genes were detected in control samples, supporting the hypothesis that swine feedlot wastewater may be a source of PMQR genes in the surrounding environment. These genes could have migrated along with quinolone-resistant bacteria and horizontally mobile genetic elements and transferred from swine feedlots to agricultural fields during agricultural applications of swine waste and wastewater. The significant positive correlation between PMQR genes in paired wastewater and soil samples further supports the possibility that swine feedlots are sources of PMQR gene contamination in adjacent farm fields.

Some (fluoro)quinolone residues were also commonly detected among the swine wastewater samples, probably reflecting their frequent usage in swine feeding practices. The wastewater concentrations among the different swine feedlots varied by about two orders of magnitude, which could be related to differences in antibiotics usage and operational scales of the swine feedlots.

Significant correlations were found between some individual (fluoro)quinolones and individual PMQR genes as well as between total (fluoro)quinolones and PMQR genes among all the paired samples, which is consistent with the hypothesis that exposure to antibiotics could lead to selective pressure for resistance genes (Wu et al. 2010). However, the correlations were not as strong as those found in other studies (Smith et al. 2004), which may reflect a variation in the fate and transport of resistance genes and antibiotics after their release into the environment (Peak et al. 2007).

Swine wastewater that contains PMQR genes and (fluoro)quinolone residues and is applied to agricultural fields or released to surrounding rivers might increase the risk that nearby residents will be exposed during farming or through their use of contaminated river water. Our sampling campaign coincided with the rainy season, and thus some of the field-applied swine waste could have been transferred by rain and wind to surrounding rivers and other environmental compartments. Sapkota et al. (2007) suggested that resistant bacteria in surface water sources contaminated by swine waste could contribute to the spread of antibiotic resistance in humans and the environment. In addition, Wilcks et al. (2004) confirmed that antibiotic resistance genes could be transferred between agricultural fields and plants that could enter into the human food cycle. During our sampling campaign we often observed young children playing in the river around the swine feedlots, probably increasing their risk of exposure. In addition, local residents informed us that they used to wash vegetables or fruit in the contaminated river. Rural environments and life styles might thus increase the risk of exposure to water and soil contaminated by PMQR genes and (fluoro)quinolones and could have health implications for local residents.

There were several limitations to our study. The swine producers declined to provide us with antibiotic usage data for proprietary reasons, thus, we chose to analyze the samples for some commonly used (fluoro)quinolones. A more extensive sampling campaign including crops, waste from humans, and additional river water samples collected at different distances from the point sources would permit a more detailed assessment of the environmental health risk of PMQR genes. In addition, phylotype and phylogenetic analyses should be conducted in future studies to track the fate of specific PMQR genes from swine feedlots to the environment.

## Conclusions

To our knowledge, this is the first study to examine PMQR genes in environmental samples collected from swine production facilities using a culture-independent method. It is also, to our knowledge, the first report on the occurrence of *qnr*D, *oqx*A, and *oqx*B in environmental samples. Our findings are consistent with the hypothesis that the PMQR genes found in fields adjacent to swine feedlots were transported from the feedlots through waste amendment and irrigation, and highlight the potential role of swine feedlots as a source of antibiotic resistance genes identified in other environment compartments. The correlations observed between PMQR genes and (fluoro)quinolone residues in soil and wastewater samples would also be consistent with the positive selection of antibiotic resistance genes as a consequence of antibiotic residues in the environment. Therefore, selection for PMQR genes could occur both in the animal gut (as a result of feeding practices) and after the environmental release of (fluoro)quinolones.

The rapid expansion of swine production and its potential role as a source of PMQR genes in the environment highlights the importance of international cooperation to promote the prudent use of antibiotics in medical therapy, agriculture, and animal husbandry and supports the need for effective treatment of husbandry wastewater before its release into the environment. The correlation of PMQR genes between wastewater and paired farm soil is a valuable first step in the environmental risk assessment of PMQR genes, but further research is needed to better understand transfer mechanisms. We also recommend the establishment of programs to monitor antibiotic resistance genes in the environment on a global scale in order to clarify the extent of potential risks to public health.

## Supplemental Material

(459 KB) PDF
