# Supplementary material for: Plasmid-Mediated Quinolone Resistance Genes and Antibiotic Residues in Wastewater and Soil Adjacent to Swine Feedlots: Potential Transfer to Agricultural Lands
Source: Environ Health Perspect. 2012 May 8;120(8):1144–9. doi: 10.1289/ehp.1104776 (PMC3440090; doi:10.1289/ehp.1104776)
Supplement: (459 KB) PDF [file ehp.1104776.s001.pdf]

Supplemental Material

Plasmid-Mediated Quinolone Resistance Genes and Antibiotic Residues in Wastewater and Soil Adjacent to Swine Feedlots: Potential Transfer to Agricultural Lands

Juan LI, Thanh WANG; Bing SHAO, Jianzhong SHEN, Shaochen WANG, Yongning WU

Table of Contents

Technical Details of Relevant Methods 2

PCR assays for PMQR genes. 2

QPCR assays for PMQR genes 2

SPE-UPLC-MS/MS. 3

Supplemental Material, Figure S1: Typical gel images of amplified PMQR genes represented here by *oqx*B (131 bp) and *oqx*A (392 bp) genes 5

Supplemental Material, Table S1: PCR (QPCR) primers and conditions used in this study 6

Supplemental Material, Table S2: PCR detection results of PMQR genes in total DNA 7

Supplemental Material, Table S3: Absolute concentrations of PMQR genes and 16S rRNA in this study, normalized to sample volume or mass 8

Supplemental Material, Table S4: Relative abundances of PMQR genes in this study, normalized to corresponding 16S rRNA copies 9

Supplemental Material, Table S5: Concentrations of (fluoro)quinolone residues in this study, normalized to sample volume or mass. 10

Supplemental Material, Table S6: Correlations between relative abundances of PMQR genes and (fluoro)quinolone residues in this study 11

References 12

Technical Details of Relevant Methods

***PCR assays for PMQR genes*:** Qualitative PCR assays were used to assess the presence of PMQR genes: *qnr*A, *qnr*B, *qnr*C, *qnr*D, *qnr*S, *qep*A, *oqx*A, *oqx*B and *aac(6')-Ib-cr*. The previously designed and validated specific primers and products length are listed in Supplemental Material, Table S1. Amplifications were conducted in 20 μL reaction volume containing 0.5 μL template DNA, 0.2 μL 10 nmol/L of each primer, 10 μL 2×PCR Mix (TianGen, China) and 9.1 μL ddH_2O, using an PCR instrument (Bio-Rad, U.S.). For amplification of the different amplicons, appropriate program parameters were used. The thermal cycle was initially denaturated at 95 °C for 5 min, followed by 35 cycles of 94 ℃ for 45 s, annealing for 45 s at different temperatures (Supplemental Material, Table S1), then 72 °C (with the exception of *oqx*A: 68 °C) for 1 min, with a final extension of 72 °C for 10 min, and then stored at 4 °C.

***QPCR assays for PMQR genes*:** To minimize qPCR inaccuracy caused by potential difference in amplification efficiency between the standard and target (Chen et al. 2010; Yu et al. 2005), the abundance of each gene was quantified against its respective sample-derived standard using the respective specific primers in Supplemental Material, Table S1 which were the same as those used in qualitative PCR, synthesized by Invitrogen. These sample-derived standards from positive samples of corresponding genes were generated as described above, and diluted serially in molecular biology-grade water. Typically, DNA template (1 μL), optimized quantities (0.2 μL) of forward and reverse primers (Invitrogen, U.S.) were combined

with MightyAmp™ for Real Time (SYBR® Plus) mix (TaKaRa, China) and molecular biology-grade water to create 20 μL reaction volumes. Optimal qPCR conditions were determined empirically, consisting of 10 min initial denaturation at 95 °C, followed by 40 cycles of denaturation at 95 °C for 15 s, and anneal (detailed in Supplemental Material, Table S1) and extension at 72 °C (with the exception of *oqx*A: 68 °C) for 1 min, with reading plate after each cycle. The qPCR reactions for 16S rRNA were performed in the same reaction mixtures as the PMQR genes, with a temperature program of 15 min at 95 °C, followed by 40 cycles of the following: 20 s at 95 °C, 45 s at 62 °C, and 30 s at 72 °C, with reading plate after each cycle.

***SPE-UPLC-MS/MS*：** In this study, ten (fluoro)quinolones (ofloxacin, ciprofloxacin, norfloxacin, enrofloxacin, lomefloxacin, pefloxacin, marbofloxacin, pipemidic acid, danofloxacin and orbifloxacin, St. Louis, MO, USA, >97%) in wastewater, soil and control samples were analyzed by a Waters Acquity LC™ system coupled with Premier mass spectrometer (Waters, Milford, MA, USA) after solid-phase extraction (SPE) preparation.

Each freeze-dried and homogenized soil sample (2-5 g) was weighed into 50-mL polyethylene centrifuge tube. 10-mL ammonia/acetonitrile/water (v/v/v, 2:25:37) solution was added and vigorously vortexed for about 30 s, which was then ultrasonicated in water bath for 15 min and finally centrifuged at 10000 rpm for 10 min. The supernatant was decanted, and the extraction procedure was repeated again. The two portions of supernatant were combined and diluted to 200 mL with water. About 0.5 g EDTA-Na_2 was added to above solution. After dissolving, the solution

was passed through an Oasis® HLB cartridge (6cc, 150 mg, Waters) which was pre-treated in sequence with 5 mL methanol and 5 mL water at a rate of about 5 mL/min. Frozen wastewater samples (about 500 mL) were thawed and pre-filtered with Whatman GF/A filter paper (Whatman, U.K.) before addition of 0.5 g EDTA-Na_2, followed by passing through an Oasis® HLB cartridge (6cc, 150 mg, Waters). After sample loading, the cartridge was washed with 5 mL water, and then thoroughly dried in vacuum for 10 min. The Sep-Pak® NH_2 cartridge (6cc, 500 mg, Waters) pre-treated with 5 mL acetone was combined with HLB cartridges in series. The sample solution was loaded onto the Oasis® HLB cartridge, washed with 5 mL of 0.1 % formic acid in methanol and further washed from the Sep-Pak® NH_2 cartridge successively with 5 mL of 0.1 % formic acid mixed with 99.9 % methanol/ acetone (1:1 v/v) and 5 mL of 0.1 % formic acid in acetone. The final eluate (about 15 mL) was evaporated to dryness by a gentle nitrogen stream and finally made up to 1 mL with the initial mobile phase (methanol–water, 5:95, v/v) for LC-MS/MS analysis.

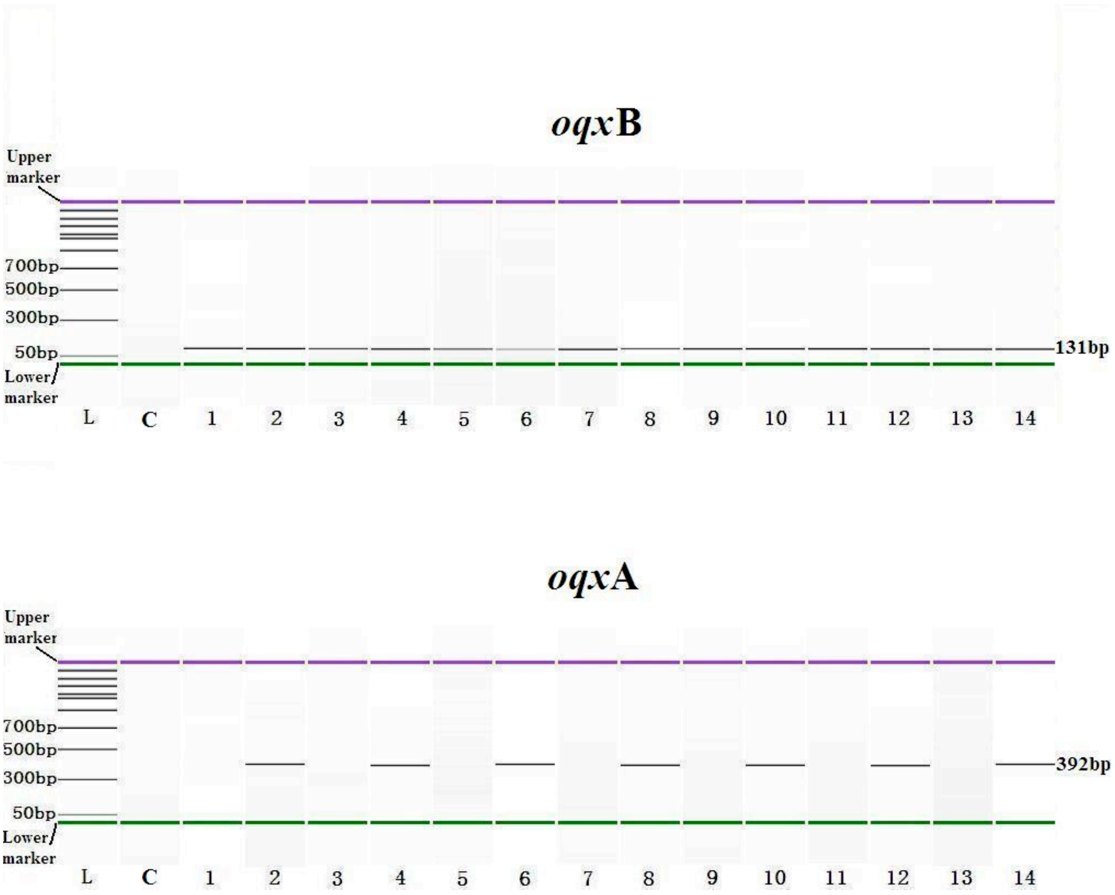

Supplemental Material, Figure S1: Typical gel images of amplified PMQR genes represented here by *oqx*B (131 bp) and *oqx*A (392 bp) genes. Each lanes represents, from left to right: (L) DNA ladder; (C) Control sample for site S (soil) (1) Sample S-s; (2) S-w; (3) F_1-s; (4) F_1-w; (5) F_2-s; (6) F_2-w; (7) F_3-s; (8) F_3-w; (9) D_1-s; (10) D_1-w; (11) D_2-s; (12) D_2-w; (13) D_3-s; (14) D_3-w. All other control samples were also negative.

Supplemental Material, Table S1: PCR (QPCR) primers and conditions used in this study.

Target gene	Primer sequence (5'-3')	Amplicon size (bp)	Annealing temperature (°C)	Reference
*qnr*A	F: AGAGGATTTCTCACGCCAGG<br>R: TGCCAGGCACAGATCTTGAC	580	54	(Cattoir et al. 2007a)
*qnr*B	F: GGMATHGAAATTCGCCACTG<br>R: TTTGCYGYYCGCCAGTCGAA	264	54	(Cattoir et al. 2007b)
*qnr*C	F: GGGTTGTACATTTATTGAATCG<br>R: CACCTACCCATTTATTTTCA	307	55	(Bin Kim et al. 2009)
*qnr*D	F: CGAGATCAATTTACGGGGAATA<br>R: AACAAGCTGAAGCGCCTG	465	50	(Cavaco et al. 2009)
*qnr*S	F: GCAAGTTCATTGAACAGGGT<br>R: TCTAAACCGTCGAGTTCGGCG	428	54	(Cattoir et al. 2007a)
*qep*A	F: CCAGCTCGGCAACTTGATAC<br>R: ATGCTCGCCTTCCAGAAAA	570	60	(Xia et al. 2010)
*oqx*A	F: CTCGGCGCGATGATGCT<br>R: CCACTCTTCACGGGAGACGA	392	57	(Kim et al. 2009)
*oqx*B	F: TCCTGATCTCCATTAACGCCCA<br>R: ACCGGAACCCATCTCGATGC	131	64	(Kim et al. 2009)
aac(6')-Ib	F: TTGCGATGCTCTATGAGTGGCTA<br>R: CTCGAATGCCTGGCGTGTTT	482	58	(Park et al. 2006)
16S rRNA	799F: GGTAGTCYAYGCMSTAAACG<br>1044R: GACARCCATGCASCACCTG	263	62	(Bach et al. 2002)

Supplemental Material, Table S2: PCR detection results of PMQR genes in total DNA.

Sample			PMQR gene								
Location[a]		Sample ID	*qnr*A	*qnr*B	*qnr*C	*qnr*D	*qnr*S	*qep*A	*oqx*A	*oqx*B	*aac(6')-lb-cr*
Shunyi	Wastewater[b]	S-w	−[d]	−	−	+[e]	+	+	+	+	−
Fangshan		F_1-w	−	−	−	+	+	+	+	+	−
Fangshan		F_2-w	−	−	−	+	+	+	+	+	−
Fangshan		F_3-w	−	+	−	+	+	+	+	+	−
Daxing		D_1-w	−	−	−	+	+	+	+	+	−
Daxing		D_2-w	−	+	−	+	+	+	+	+	−
Daxing		D_3-w	−	+	−	+	+	+	+	+	−
Shunyi	Soil[c]	S-s	−	−	−	+	−	+	−	+	−
Fangshan		F_1-s	−	−	−	+	−	+	−	+	−
Fangshan		F_2-s	−	−	−	+	−	+	−	+	−
Fangshan		F_3-s	−	−	−	+	−	+	−	+	−
Daxing		D_1-s	−	−	−	+	−	+	−	+	−
Daxing		D_2-s	−	−	−	+	−	+	−	+	−
Daxing		D_3-s	−	−	−	+	−	+	−	+	−

[a] Sampling locations were located in three districts of Beijing

[b] Wastewater samples were collected from effluents from seven swine feedlots.

[c] Soil samples were collected from seven agriculture fields adjacent to corresponding swine feedlots

[d] −: negative result

[e] +: positive result

Note: all results from control samples were negative.

Supplemental Material, Table S3: Absolute concentrations[a] of PMQR genes and 16S rRNA in this study, normalized to sample volume[b] or mass[c].

Sample	PMQR gene[d]						16S rRNA
	*qnr*D	*oqx*B	*qep*A	*qnr*S	*oqx*A	Sum[e]	
S-s	4.22E+06 (1.82E+05)[f]	2.42E+05 (2.63E+04)	1.19E+07 (3.90E+06)	n.d.[g]	n.d.	1.64E+07 (4.09E+06)	1.21E+07 (9.67E+05)
S-w	7.82E+06 (8.93E+04)	4.71E+05 (1.78E+04)	2.54E+06 (2.14E+05)	1.11E+07 (4.78E+06)	2.02E+06 (4.07E+05)	2.39E+07 (5.21E+06)	2.27E+06 (2.31E+05)
F_1-s	2.94E+05 (2.17E+04)	1.45E+06 (1.95E+05)	2.32E+06 (3.41E+05)	n.d.	n.d.	4.06E+06 (5.38E+05)	1.97E+07 (2.04E+06)
F_1-w	6.04E+06 (2.25E+05)	8.07E+06 (2.91E+05)	2.53E+06 (2.14E+05)	2.06E+06 (2.76E+05)	7.66E+05 (2.24E+04)	1.66E+07 (5.24E+05)	1.85E+07 (3.21E+05)
F_2-s	7.20E+06 (2.63E+05)	3.00E+06 (3.23E+05)	9.92E+06 (2.51E+06)	n.d.	n.d.	2.01E+07 (2.53E+06)	1.98E+07 (9.71E+05)
F_2-w	8.77E+06 (9.46E+04)	4.41E+07 (2.31E+06)	2.95E+07 (3.04E+06)	1.29E+07 (2.06E+06)	1.59E+06 (1.65E+05)	9.69E+07 (5.96E+06)	1.87E+07 (1.13E+06)
F_3-s	4.69E+05 (1.17E+04)	1.77E+06 (1.58E+05)	2.34E+07 (2.85E+06)	n.d.	n.d.	2.56E+07 (2.89E+06)	1.70E+07 (1.07E+06)
F_3-w	5.23E+06 (8.50E+04)	1.36E+07 (2.03E+06)	3.79E+07 (5.80E+06)	2.35E+07 (1.66E+06)	1.18E+06 (1.74E+05)	8.14E+07 (4.57E+06)	1.68E+07 (1.26E+06)
D_1-s	2.00E+06 (1.58E+05)	1.24E+05 (1.42E+04)	2.65E+06 (2.59E+05)	n.d.	n.d.	4.77E+06 (4.02E+05)	1.00E+07 (1.65E+06)
D_1-w	8.32E+06 (2.00E+05)	2.12E+06 (4.16E+05)	8.97E+06 (5.30E+05)	1.42E+07 (1.97E+06)	5.62E+05 (2.26E+04)	3.36E+07 (2.03E+06)	1.92E+07 (2.63E+06)
D_2-s	4.22E+06 (5.19E+04)	7.86E+07 (3.47E+06)	1.24E+07 (1.26E+06)	n.d.	n.d.	9.52E+07 (2.31E+06)	1.17E+07 (7.64E+05)
D_2-w	1.63E+07 (7.80E+05)	2.61E+08 (1.97E+07)	9.29E+07 (3.35E+06)	3.44E+07 (2.25E+06)	1.92E+06 (1.68E+05)	4.06E+08 (2.12E+07)	3.52E+07 (3.47E+06)
D_3-s	8.15E+06 (8.89E+04)	3.40E+07 (3.04E+06)	1.40E+07 (1.51E+06)	n.d.	n.d.	5.61E+07 (4.13E+06)	1.25E+07 (1.09E+06)
D_3-w	3.14E+07 (1.43E+06)	2.06E+08 (4.18E+07)	9.64E+07 (1.84E+07)	1.98E+07 (2.77E+06)	1.50E+06 (2.24E+05)	3.55E+08 (6.33E+07)	3.16E+07 (1.51E+06)

[a] All values are presented as the arithmetic means of three replicates determined by qPCR

[b] Units are copies per milliliter for wastewater samples (copies/mL)

[c] Units are copies per gram for soil samples (copies/g)

[d] *qnr*A, *qnr*B, *qnr*C and *aac(6')-Ib-cr* were not detected

[e] Sum of five PMQR genes (*qnr*D, *oqx*B, *qep*A, *qnr*S and *oqx*A)

[f] Standard errors of the means are presented in brackets

[g] n.d.: not detected

Supplemental Material, Table S4: Relative abundances[a] of PMQR genes[b] in this study, normalized to corresponding 16S rRNA copies.

Sample	PMQR gene copies/16S rRNA copies					
	*qnr*D	*oqx*B	*qep*A	*qnr*S	*oqx*A	Sum[c]
S-s	0.357 (0.047)[d]	0.021 (0.004)	1.05 (0.438)	n.d.[e]	n.d.	1.43 (0.487)
S-w	3.52 (0.357)	0.211 (0.020)	1.13 (0.104)	4.57 (1.61)	0.879 (0.107)	10.3 (1.21)
F_1-s	0.015 (0.002)	0.078 (0.019)	0.124 (0.032)	n.d.	n.d.	0.217 (0.054)
F_1-w	0.327 (0.008)	0.437 (0.022)	0.137 (0.014)	0.112 (0.017)	0.041 (0.002)	1.06 (0.048)
F_2-s	0.365 (0.014)	0.150 (0.009)	0.501 (0.118)	n.d.	n.d.	1.02 (0.110)
F_2-w	0.473 (0.031)	2.36 (0.024)	1.57 (0.101)	0.694 (0.112)	0.085 (0.008)	5.18 (0.189)
F_3-s	0.028 (0.002)	0.105 (0.012)	1.37 (0.082)	n.d.	n.d.	1.50 (0.075)
F_3-w	0.315 (0.026)	0.801 (0.057)	2.32 (0.487)	1.40 (0.053)	0.070 (0.005)	4.91 (0.496)
D_1-s	0.208 (0.030)	0.014 (0.004)	0.281 (0.059)	n.d.	n.d.	0.503 (0.093)
D_1-w	0.450 (0.064)	0.116 (0.027)	0.485 (0.073)	0.798 (0.230)	0.030 (0.003)	1.88 (0.383)
D_2-s	0.365 (0.030)	6.74 (0.214)	1.08 (0.189)	n.d.	n.d.	8.19 (0.409)
D_2-w	0.467 (0.023)	7.60 (1.06)	2.70 (0.338)	0.998 (0.119)	0.056 (0.010)	11.8 (1.47)
D_3-s	0.662 (0.060)	2.81 (0.529)	1.15 (0.201)	n.d.	n.d.	4.63 (0.773)
D_3-w	0.995 (0.024)	6.43 (0.990)	3.02 (0.447)	0.623 (0.067)	0.047 (0.005)	11.1 (1.44)

[a] All values are presented as the arithmetic means of three replicates determined by qPCR

[b] *qnr*A, *qnr*B, *qnr*C and *aac(6')-Ib-cr* were not detected

[c] Sum of five PMQR genes (*qnr*D, *oqx*B, *qep*A, *qnr*S and *oqx*A)

[d] Standard errors of the means are presented in brackets

[e] n.d.: not detected

Supplemental Material, Table S5: Concentrations[a] of (fluoro)quinolone residues in this study, normalized to sample volume[b] or mass[c].

(Fluoro)quinolones[d]	Sample													
	Wastewater[e]							Soil[f]						
	S-w	F_1-w	F_2-w	F_3-w	D_1-w	D_2-w	D_3-w	S-s	F_1-s	F_2-s	F_3-s	D_1-s	D_2-s	D_3-s
OFX	52.9 (1.56)[h]	13.0 (1.48)	0.810 (0.290)	111 (2.69)	4.57 (0.330)	39.5 (2.26)	5.85 (0.140)	5.31 (0.500)	n.d.[i]	n.d.	n.d.	n.d.	1.71 (0.200)	n.d.
CIP	176 (2.57)	9.62 (0.230)	21.0 (2.00)	6.85 (0.330)	n.d.	14.9 (0.610)	244 (2.04)	7.15 (0.170)	n.d.	n.d.	n.d.	n.d.	n.d.	20.4 (0.870)
NOR	40.6 (1.42)	38.1 (1.30)	34.3 (2.19)	7.80 (0.210)	n.d.	28.0 (1.01)	5.15 (0.310)	6.42 (0.250)	n.d.	1.16 (0.430)	n.d.	n.d.	1.40 (0.260)	1.35 (0.210)
ENRO	5.11 (0.490)	5.61 (0.310)	1.11 (0.410)	n.d.	n.d.	n.d.	24.1 (0.760)	1.30 (0.430)	n.d.	n.d.	n.d.	n.d.	n.d.	0.510 (0.060)
LOM	7.31 (0.550)	7.91 (0.470)	1.64 (0.280)	n.d.	n.d.	n.d.	9.82 (0.600)	n.d.	n.d.	n.d.	n.d.	n.d.	n.d.	1.11 (0.470)
PEFX	1.42 (0.110)	n.d.	1.40 (0.070)	n.d.	n.d.	n.d.	n.d.	n.d.	n.d.	n.d.	n.d.	n.d.	n.d.	n.d.
MB	9.72 (0.600)	n.d.	n.d.	n.d.	n.d.	n.d.	n.d.	n.d.	n.d.	n.d.	n.d.	n.d.	n.d.	n.d.
PPA	7.81 (0.300)	n.d.	n.d.	n.d.	n.d.	n.d.	n.d.	n.d.	n.d.	n.d.	n.d.	n.d.	n.d.	n.d.
DANO	12.6 (0.190)	n.d.	n.d.	n.d.	n.d.	n.d.	n.d.	n.d.	n.d.	n.d.	n.d.	n.d.	n.d.	n.d.
OBFX	7.32 (0.330)	n.d.	n.d.	n.d.	n.d.	n.d.	n.d.	n.d.	n.d.	n.d.	n.d.	n.d.	n.d.	n.d.
Total[g]	321 (2.01)	74.2 (3.53)	60.3 (4.39)	125 (3.09)	4.57 (0.330)	82.4 (3.67)	289 (3.57)	20.2 (1.13)	n.d.	1.16 (0.430)	n.d.	n.d.	3.11 (0.300)	23.4 (1.50)

[a] All values are presented as the arithmetic means of three replicates determined by SPE-UPLC-MS/MS

[b] Units are nanogram per milliliter for wastewater samples (ng/mL)

[c] Units are nanogram per gram for soil samples (ng/g)

[d] OFX=ofloxacin, CIP=ciprofloxacin, NOR=norfloxacin, OBFX=orbifloxacin, LOM=lomefloxacin, PEFX=pefloxacin, MB=marbofloxacin, PPA=pipemidic acid, DANO=danofloxacin, ENRO=enrofloxacin

[e] Wastewater samples were collected from seven swine feedlots effluent

[f] Soil samples were collected from seven agriculture fields adjacent to corresponding swine feedlots

[g] Sum of ten (fluoro)quinolones (OFX, CIP, NOR, OBFX, LOM, PEFX, MB, PPA, DANO and ENRO)

[h] Standard errors of the means are presented in brackets

[i] n.d.: not detected

Supplemental Material, Table S6: Correlations[a] between relative abundances[b] of PMQR genes and (fluoro)quinolone residues in this study.

(Fluoro)quinolones[c]	PMQR gene					
	*qnr*D	*oqx*B	*qep*A	*qnr*S	*oqx*A	Sum[d]
OFX	0.31 (0.28)	-0.01 (0.98)	0.45 (0.11)	***0.58 (0.03)***	0.39 (0.16)	0.35 (0.22)
CIP	***0.70 (0.01)***	0.30 (0.30)	0.50 (0.07)	***0.56 (0.04)***	***0.56 (0.04)***	***0.65 (0.01)***
NOR	***0.54 (0.04)***	0.08 (0.79)	0.14 (0.64)	***0.59 (0.03)***	***0.60 (0.02)***	0.40 (0.16)
ENRO	0.30 (0.29)	0.37 (0.19)	0.48 (0.08)	0.15 (0.62)	0.14 (0.63)	0.47 (0.09)
LOM	***0.57 (0.03)***	0.15 (0.60)	0.22 (0.45)	0.42 (0.14)	0.47 (0.09)	0.42 (0.14)
PEFX	n/a[f]	n/a	n/a	n/a	n/a	n/a
MB	n/a	n/a	n/a	n/a	n/a	n/a
PPA	n/a	n/a	n/a	n/a	n/a	n/a
DANO	n/a	n/a	n/a	n/a	n/a	n/a
OBFX	n/a	n/a	n/a	n/a	n/a	n/a
Total[e]	***0.80 (0.001)***	0.23 (0.42)	***0.54 (0.04)***	***0.77 (0.001)***	***0.73 (0.003)***	***0.71 (0.005)***

[a] Values indicate the Pearson correlation coefficient (r), and the p-values are presented in brackets. The bold values in italics indicate statistical significance ($p < 0.05$).

[b] The absolute copies of PMQR genes normalized to corresponding 16S rRNA copies

[c] OFX=ofloxacin, CIP=ciprofloxacin, NOR=norfloxacin, OBFX=orbifloxacin, LOM=lomefloxacin, PEFX=pefloxacin, MB=marbofloxacin, PPA=pipemidic acid, DANO=danofloxacin, ENRO=enrofloxacin

[d] Sum of five PMQR genes (*qnr*D, *oqx*B, *qep*A, *qnr*S and *oqx*A)

[e] Sum of ten (fluoro)quinolones (OFX, CIP, NOR, OBFX, LOM, PEFX, MB, PPA, DANO and ENRO)

[f] n/a: no correlation available since PEFX, MB, PPA, DANO and OBFX were only detected in two or one sample

References

Bach HJ, Tomanova J, Schloter M, Munch JC. 2002. Enumeration of total bacteria and bacteria with genes for proteolytic activity in pure cultures and in environmental samples by quantitative PCR mediated amplification. J Microbiol Meth 49:235-245.

Bin Kim H, Park CH, Kim CJ, Kim EC, Jacoby GA, Hooper DC. 2009. Prevalence of plasmid-mediated quinolone resistance determinants over a 9-year period. Antimicrob Agents Chemother 53:639-645.

Cattoir V, Poirel L, Rotimi V, Soussy CJ, Nordmann P. 2007a. Multiplex PCR for detection of plasmid-mediated quinolone resistance *qnr* genes in ESBL-producing *enterobacterial* isolates. J Antimicrob Chemother 60:394-397.

Cattoir V, Weill FX, Poirel L, Fabre L, Soussy CJ, Nordmann P. 2007b. Prevalence of *qnr* genes in *Salmonella* in France. J Antimicrob Chemother 59:751-754.

Cavaco LM, Hasman H, Xia S, Aarestrup FM. 2009. *qnr*D, a novel gene conferring transferable quinolone resistance in *Salmonella enterica* serovar Kentucky and Bovismorbificans strains of human origin. Antimicrob Agents Chemother 53:603-608.

Chen J, Michel FC, Sreevatsan S, Morrison M, Yu ZT. 2010. Occurrence and persistence of erythromycin resistance genes (*erm*) and tetracycline resistance genes (*tet*) in waste treatment systems on swine farms. Microb Ecol 60:479-486.

Kim HB, Wang M, Park CH, Kim EC, Jacoby GA, Hooper DC. 2009. *oqx*AB encoding a multidrug efflux pump in human clinical isolates of

Enterobacteriaceae. Antimicrob Agents Chemother 53:3582-3584.

Park CH, Robicsek A, Jacoby GA, Sahm D, Hooper DC. 2006. Prevalence in the United States of *aac(6')-Ib-cr* encoding a ciprofloxacin-modifying enzyme. Antimicrob Agents Chemother 50:3953-3955.

Xia LN, Li L, Wu CM, Liu YQ, Tao XQ, Dai L, et al. 2010. A survey of plasmid-mediated fluoroquinolone resistance genes from *Escherichia coli* isolates and their dissemination in Shandong, China. Foodborne Pathog Dis 7:207-215.

Yu ZT, Michel FC, Hansen G, Wittum T, Morrison M. 2005. Development and application of real-time PCR assays for quantification of genes encoding tetracycline resistance. Appl Environ Microbiol 71:6926-6933.
